# CO_2_ capture technology based on gas hydrate method: a review

**DOI:** 10.3389/fchem.2024.1448881

**Published:** 2024-10-17

**Authors:** Jialing Pei, Jinger Chen, Jingxue Wang, Zhi Li, Nan Li, Jingyu Kan

**Affiliations:** State Key Laboratory of Heavy Oil Processing, China University of Petroleum-Beijing at Karamay, Karamay, China

**Keywords:** hydrate, capture of CO_2_, separation of gas mixture, chemical promoter of hydrate, mechanical promotion of hydrate

## Abstract

At present, the problem of global warming is becoming increasingly serious, and one of the main culprits is the increasing amount of carbon dioxide emissions. Although the traditional carbon capture technologies can reduce the concentration of CO_2_ in the atmosphere, it has a series of problems such as high energy consumption, high cost, low efficiency or unfriendly environment. Hydrate-based carbon dioxide separation are considered to be a technology with great application and development prospects. Compared with the traditional method of carbon dioxide separation, the hydrate method has the advantages of simple process, low energy consumption and environmental friendliness. This review introduces the advantages of hydrate method compared with traditional carbon capture technologies, expounds the theory of carbon dioxide capture by hydrate, and the strengthening and improvement techniques of hydrate method, including thermodynamic promoter, kinetic promoter and mechanical reinforcement, and introduces the practical application of hydrate method in various fields.

## 1 Introduction

### 1.1 Research background

The emission of greenhouse gases caused by human activities is the main cause of global temperature rise, and global warming seriously threatens human survival. Carbon dioxide is one of the main gases that cause the greenhouse effect. According to the statistics and predictions of the United Nations Intergovernmental Panel on Climate Change (IPPC), Carbon dioxide emissions from human activities will reach 35.8 billion tons in 2023. By 2,100, the concentration of carbon dioxide in the atmosphere may reach 570 ppm, which will cause the global average temperature to rise by about 1.9°C (Xie et al., 2019). Therefore, controlling the emission of greenhouse gases has become an urgent matter for all countries in the world. To prevent the exacerbation of the greenhouse effect, a series of clean energy sources, e.g., solar energy, wind energy, and biomass fuels, are being developed as substitutes for fossil fuels. However, these clean energy sources cannot yet replace fossil energy to meet our needs. Fossil energy will still be the main energy source for our daily life and industrial production in the future. Therefore, further research and development on carbon dioxide capture, separation and storage will be a vital method to address current development and environmental issues.

### 1.2 Capture of carbon dioxide

The capture of carbon dioxide refers to the process of capturing and separating carbon dioxide from the gas mixture, and then store it permanently, thereby reducing the content of carbon dioxide in the atmosphere (Li, 2014). The separation of carbon dioxide is generally divided into three categories: pre-combustion capture, post-combustion capture, and oxyfuel combustion capture. Currently, the most widely used method in industry is post-combustion. The traditional carbon dioxide capture methods mainly include physical adsorption, absorption and membrane separation technology (Madejski et al., 2022).

#### 1.2.1 Adsorption

Gas adsorption include physical adsorption and chemical adsorption. There are two kinds of physical adsorption methods: pressure swing adsorption and temperature swing adsorption (Zhang, 2014). The main difference between the two is: one is to change the pressure, and the other is to change the temperature. Moreover, temperature swing adsorption is considered to be more suitable for post-combustion CO_2_ capture (Gutierrez-Ortega et al., 2022), while pressure swing adsorption is more suitable for pre-combustion CO_2_ capture (Abd et al., 2023). Under specific pressure and temperature conditions, solid adsorbents selectively adsorb carbon dioxide to their pores, cracks or surfaces. Common solid adsorbents include activated carbon, zeolite, etc. Activated carbon materials are low in cost and high in thermal stability. In recent years, the research on the preparation and modification of activated carbon for CO_2_ capture has gradually become a hot spot. Biomass activated carbon for CO_2_ capture mainly uses KOH as the activator. Shi and Liu (2021) synthesized two kinds of nitrogen-doped activated carbon by microwave/KOH activation and thermal/KOH activation, to separate CO_2_ from flue gas. By comparison, it was found that the nitrogen-doped activated carbon activated by microwave/KOH have developed pore structure and abundant active functional groups, resulting in high CO_2_ adsorption capacities. Owing to the strong corrosiveness, long activation time and complicated processing technology of KOH, other types of activators have been developed to replace it. Li and Xiao (2019) prepared a dual pore structure activated carbon with both micropores and mesopores from rice husk charcoal by leaching with K_2_CO_3_, and its specific surface area, micropore volume and mesopore volume were 1,097 m^2^/g, 0.34 cm^3^/g and 0.49 cm^3^/g respectively, showing excellent CO_2_ adsorption capacity and good CO_2_ selectivity.

**FIGURE 1:**
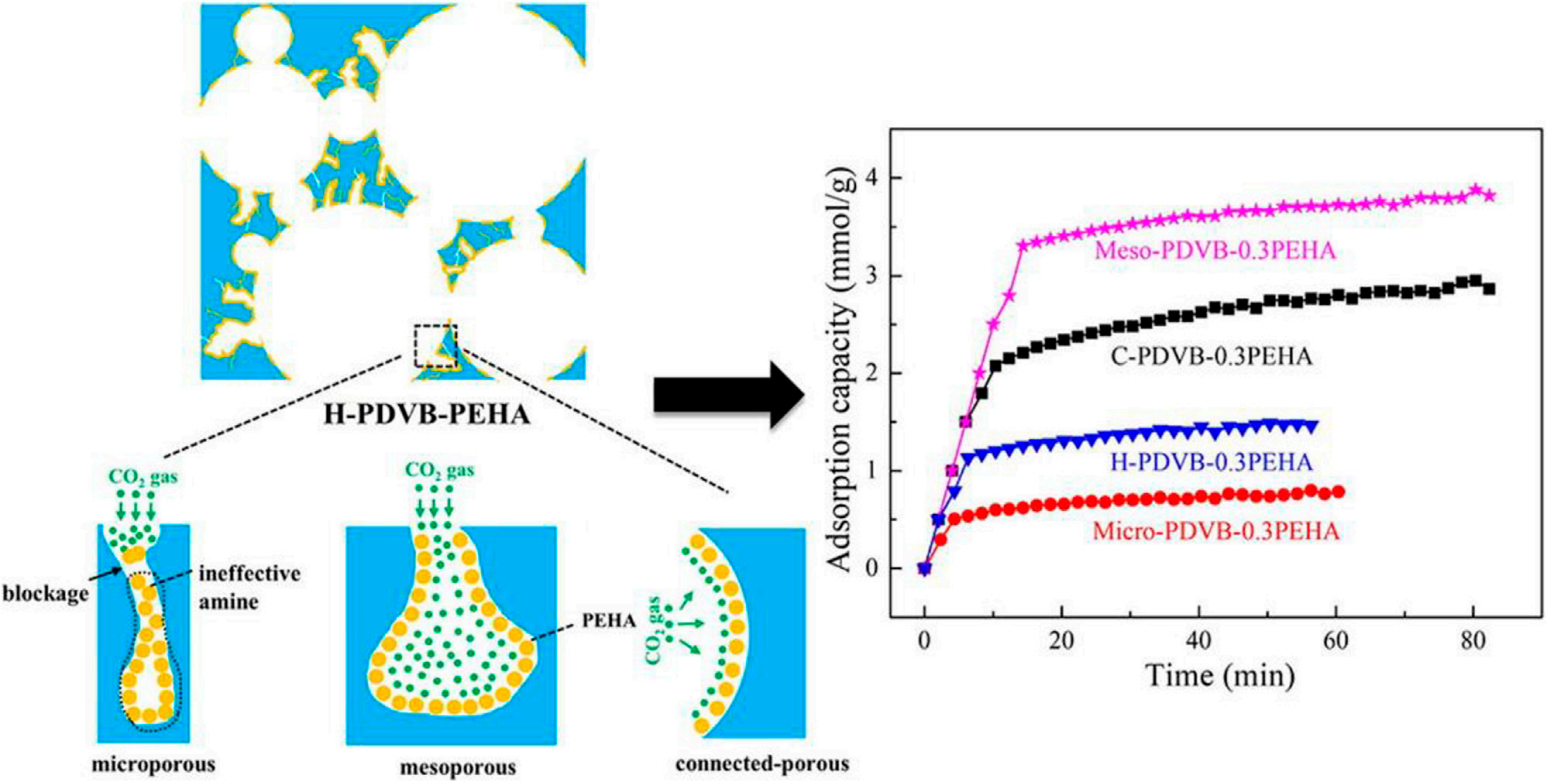
Diffusion of CO_2_ molecules in different pore structure of hierarchically porous monoliths (Lee et al., 2011).

The chemisorption of CO_2_ is achieved by forming active sites on the surface of the adsorbent to increase the selectivity of CO_2_ molecules (Lee et al., 2011). So far, the commonly used chemical adsorbents are amines and metal oxides. Solid amine adsorbents have the properties of excellent adsorption performance, simple regeneration process, and environmentally friendly equipment (Wang et al., 2022), and have good development prospects. However, the amine functionalization of most carriers may block the micropores, hinder the diffusion of CO_2_ (Figure 1), and even lead to a decrease in the pore volume and specific surface area of the adsorbent, thereby affecting the adsorption-desorption kinetics of CO_2_ (Yan et al., 2022). Compared with other chemical adsorbents, metal oxides have advantages in stability, low toxicity and high economic benefit (Azmi, Ruhaimi, and Aziz, 2020). Alkaline earth metal oxides, e.g., magnesium oxide and calcium oxide, are widely used in the CO_2_ absorption process (Khdary et al., 2022). However, since these oxides will generate some by-products during the adsorption process, the adsorption capacity of CO_2_ decreases greatly with the increase of cycle times (Cho et al., 2018).

The advantage of the adsorption method lies in the reuse of the adsorbent. But the separation of carbon dioxide by the adsorption method has higher requirements on the selectivity and capacity of the adsorbent than others, and there is also significant room for improvement in terms of separation efficiency.

#### 1.2.2 Absorption

**FIGURE 2:**
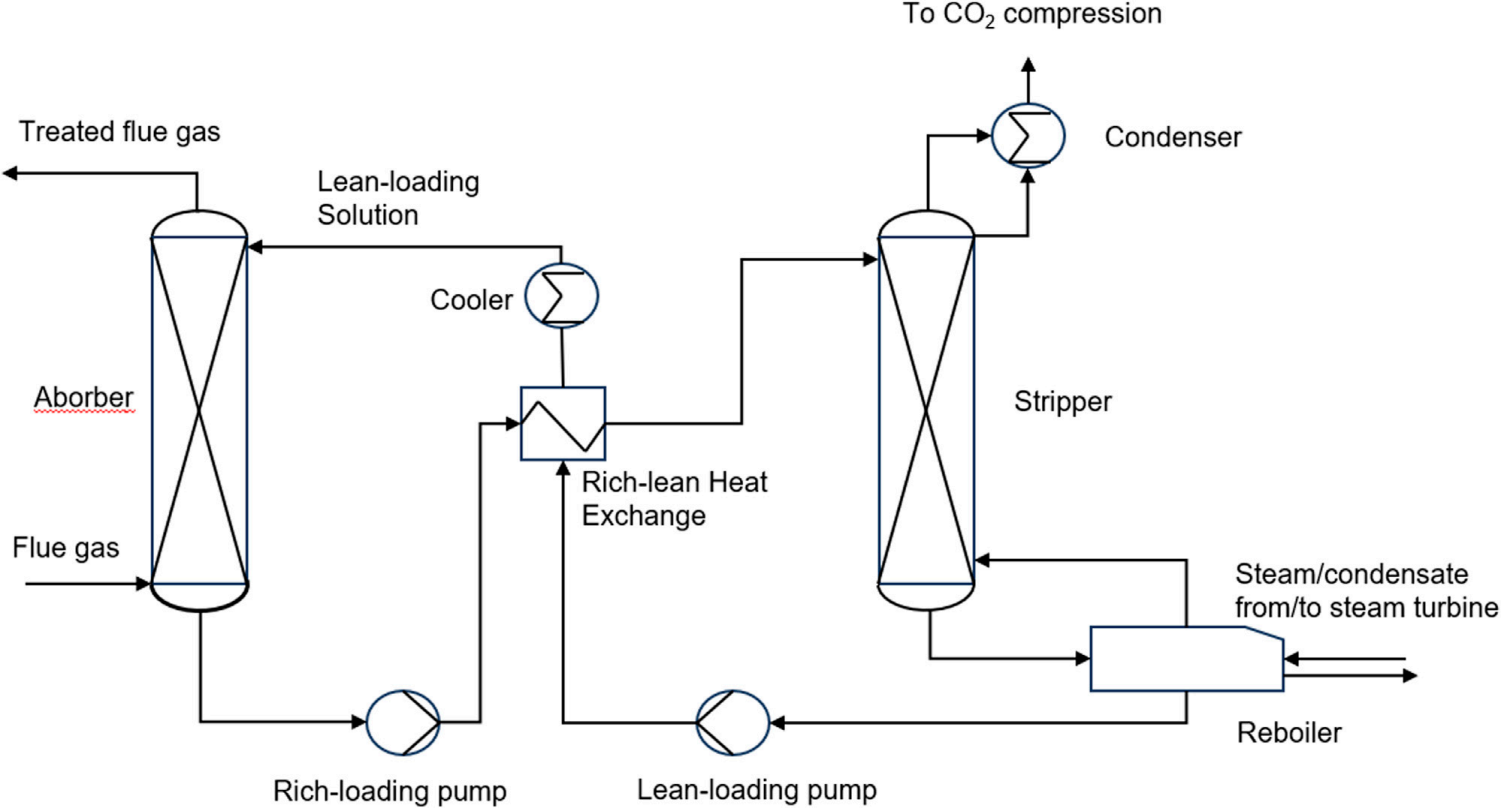
Schematic of a basic chemical absorption process for CO_2_ capture (Wang et al., 2017).

The absorption method is divided into biological absorption, physical absorption and chemical absorption. Bio-absorption refers to the use of plants to absorb carbon dioxide in the atmosphere through photosynthesis. The physical absorption method refers to the pressure dependent solubility of carbon dioxide in solvent, e.g., water, and methanol, to capture carbon dioxide (Zong et al., 2016). In recent years, ionic liquids with high molecular weight, deep eutectic solvents and some polymers with various functional groups have been proved to be satisfactory physical absorbents for CO_2_ (Li et al., 2022). Chemical absorption method refers to the use of the acidic gas properties of carbon dioxide to react with weakly alkaline liquid solvents or solid substrates to absorb and separate carbon dioxide from the gas mixture (Xiao et al., 2022). Chemical absorption could be divided into two steps (Madejski et al., 2022). In the first step, the gas mixture react with the absorbent to capture CO_2_. The second step is to transfer the reaction solution to the stripping tower to regenerate CO_2_ at high temperature (Figure 2). The advantage of the absorption method is that the solvent can be recycled and carbon dioxide gas with high purity can be obtained, but its cost is high and the solvent consumption is large. Mohamadi-Baghmolaei et al. used adsorption to remove acid gases from mixed gases (Mohamadi-Baghmolaei et al., 2021). Although good separation effect was achieved, the process energy consumption accounted for nearly half of the process cost. Changing the operating conditions and improving the process have become an urgent step for absorption method.

#### 1.2.3 Cryogenic separation technology

**FIGURE 3:**
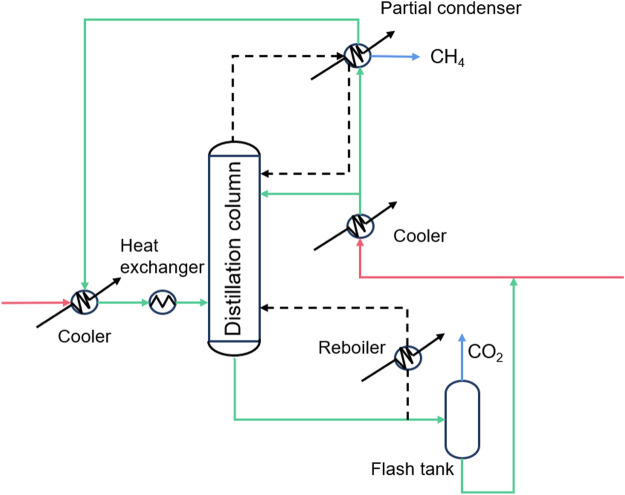
Schematic of cryogenic separation technology (Song et al., 2019).

Cryogenic separation technology is based on the difference in the phase transition properties of the components during a series of compression, cooling, and expansion processes to realize separation (Xu et al., 2012). CO_2_ cryogenic separation technology uses the characteristics of desublimation or condensation of each component of the mixed gas to separate liquid or solid CO_2_ (Figure 3). The cryogenic separation technology is relatively simple, and the separated CO_2_ gas has higher purity and easier to transport. Compared with other separation technologies, it is more environmentally friendly and non-corrosive. It can also remove other polluting gases e.g., sulfur oxides, mercury, and nitrogen oxides, etc. (Nandakishora, Sahoo, and Murugan, 2022) Cryogenic separation technology has a relatively mature industrial foundation and is easy to expand to industrial scale. However, the extremely low temperature, large energy consumption and high investment cost of equipment required for CO_2_ gas condensation process limit its development (Shen et al., 2022; Baena-Moreno et al., 2019). Zhang et al. proposed a hybrid system combining cryogenic separation carbon capture and liquid air energy storage (CS-LAES), which can increase the CO_2_ capture rate to 99.97%, but it still requires huge energy consumption. It can be seen that there is still a long way to go before cryogenic separation technology is widely used in industrialization (Zhang et al., 2024).

#### 1.2.4 Membrane separation technology

**FIGURE 4:**
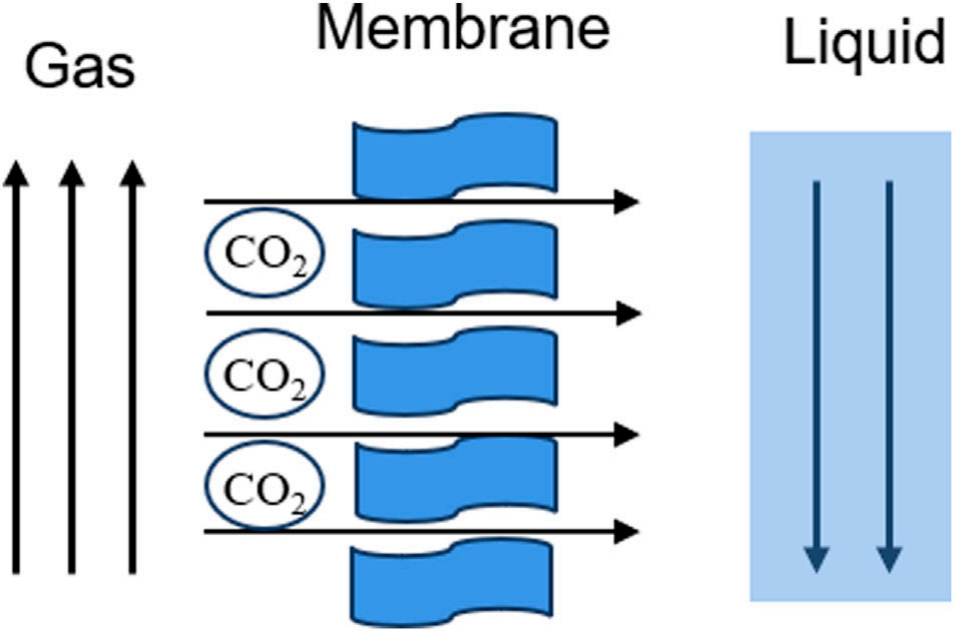
Schematic of membrane separation technology (Guo et al., 2023).

The membrane separation method is mainly based on the different diffusion rate and relative permeability of various gas molecules in the membrane material, so as to achieve the separation effect (Figure 4). During the membrane separation process, the pressure difference on both sides of the membrane is the driving force for gas molecules to pass through the membrane. When the pressure difference on both sides of the membrane reaches a certain value, the gas molecules with high relative permeability pass through the membrane preferentially (Guo et al., 2023) to realize the separation of gas mixture. Inorganic membranes, metal membranes, polymer membranes and solid-liquid membranes have been developed to separate CO_2_. The well-known gas transport mechanisms of CO_2_ separation membranes include the solution diffusion mechanism, enhanced transport mechanism, and molecular sieve mechanism. The separation of CO_2_ gas by polymer usually follows a simple solution diffusion mechanism. Because of the unique chemical structure of CO_2_ molecules, the reversible chemical reaction between base groups and CO_2_ promotes the transport of CO_2_ between membranes. This mechanism is called the promoted transport mechanism (Sandru et al., 2022). The molecular sieve mechanism means that the membrane material allows small molecules to pass through and intercepts large molecules. This mechanism usually exists in the transmission process of micro-porous membrane materials (Ma et al., 2020). PIM is a common gas separation membrane, which is widely used for CO_2_ capture in the mixed gas of coal-fired and gas-fired power plants due to its unique microporous structure (Pasichnyk et al., 2023). However, PIM is fragile and will seriously age after long-term use (Gao et al., 2021). The gas separation selectivity will also be greatly reduced. The membrane separation process is relatively simple, easy to operate, and low in energy consumption. However, the cost of membrane materials used in this process is high, and the treatment, dehydration, and filtration are usually required before the separation of carbon dioxide, and the purity of the final carbon dioxide obtained is not high.

#### 1.2.5 High-pressure supersonic separation technology

**FIGURE 5:**
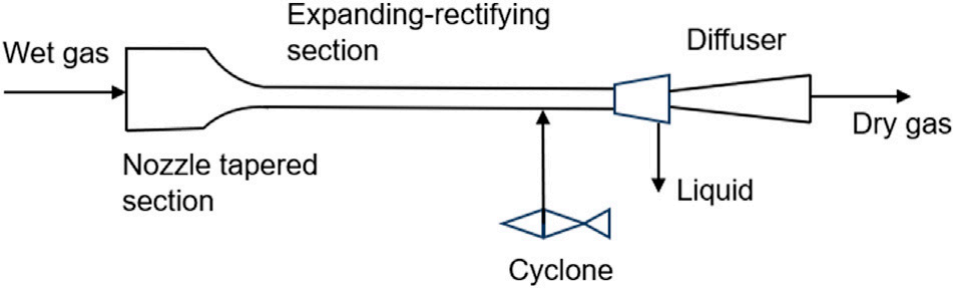
Structure of supersonic separator (Jiang et al., 2018).

Supersonic separation is a physical separation method with no pollution, low energy consumption and high efficiency (Arinelli et al., 2017). The theory of this technology is that the mixed gas is cooled and condensed into droplets through a supersonic nozzle, and then a vortex generator is used to generate a vortex to push the droplets to the wall to form a liquid film (Figure 5). The liquid film will be separated from the liquid outlet, thus reducing the proportion of condensable components in the mixture (Jiang et al., 2018). This technology provides a new idea for decarbonization, and the key of the new idea is carbon condensation in supersonic nozzles. In general, the non-equilibrium condensation characteristics in supersonic expanding flows are stronger than the coupling between the flow field and CO_2_ condensation. In recent years, some scholars have simulated the feasibility of this method through numerical simulation and other methods. The simulation results show that this method has certain feasibility, but there is still a lack of simulation and experimental research on supercritical CO_2_ (Ding et al., 2023).

#### 1.2.6 Electrochemical carbon capture technology

Electrochemical carbon capture technology is a traditional carbon capture technology that emerged in the last century (Wang F et al., 2024). At present, electrochemical carbon capture technology has been widely used in various fields, mainly through redox active carriers or pH to achieve CO_2_ capture (alkaline) and recovery (acidic) (Zhu et al., 2023). Some active carriers, e.g., quinones, can bind and release CO_2_ in the redox state to achieve CO_2_ capture and recovery. By electrochemical carbon capture technology with controlled pH, salt or water electrolysis is used to separate alkaline and acidic solutions for CO_2_ absorption. Compared with other traditional methods (absorption, adsorption, etc.), electrochemical carbon capture has advantages of higher energy efficiency, flexibility and sustainability. However, it still has shortcomings of low capture rate, and it is still in the theoretical stage. With the deepening of research and the expansion of application scope, it is expected to achieve industrial application in the future (Rahimi et al., 2022).

The use of these methods to capture carbon dioxide poses numerous problems, e.g., high energy consumption, uneconomical, unfriendly environment and low purity (Table 1). Compared with the above-mentioned traditional capture processes, the hydrate capture and storage of carbon dioxide has the advantages of simple process, low cost, and environmental friendliness.

**TABLE 1:** Various types of carbon capture technologies.

Method		Theory	Advantage	Disadvantage	References
Adsorption	physical adsorption	The physical interaction between adsorbate and adsorbent	Adsorbent can be reused	Higher requirements on the selectivity and capacity of the adsorbent, and has low separation efficiency	Shi and Liu, (2021)
	chemical adsorption	Chemical bonds are formed between adsorbate and adsorbent			Yan et al. (2022)
Absorption	biological absorption	Photosynthesis	The solvent can be recycled; the purity of the product CO_2_ is relatively high	High solvent consumption and cost	
	physical absorption	Solubility changes with pressure			Zong et al. (2016)
	chemical absorption	Acid-base reaction			Xiao et al. (2022)
Cryogenic separation		Differences in phase transition properties of components	Simple technology; the product has high purity of CO_2_; environmentally friendly	High energy consumption and cost	Xu et al. (2012)
Membrane separation		Differences in relative permeability	Easy to operate; low energy consumption	High cost and low CO_2_ purity	Guo et al. (2023)
High-pressure supersonic separation technology		CO_2_ non-equilibrium condensation and vortex separation	No pollution, low cost and high efficiency	Lack of simulations and experimental studies on supercritical CO_2_	Arinelli et al. (2017)
Electrochemical carbon capture technology		Redox active carriers or pH swing	Strong applicability	Low capture rate and still in the theoretical stage	Zhu et al. (2023)
Hydrate		Hydrate phase equilibrium principle	Large gas storage capacity, reversible process, and great development prospects	High energy consumption, low separation selectivity, hydration rate, efficiency and hydrate stability	Li et al. (2024)

## 2 Hydrate-based CO_2_ capture and separation

### 2.1 Gas hydrate and the theories

**FIGURE 6:**
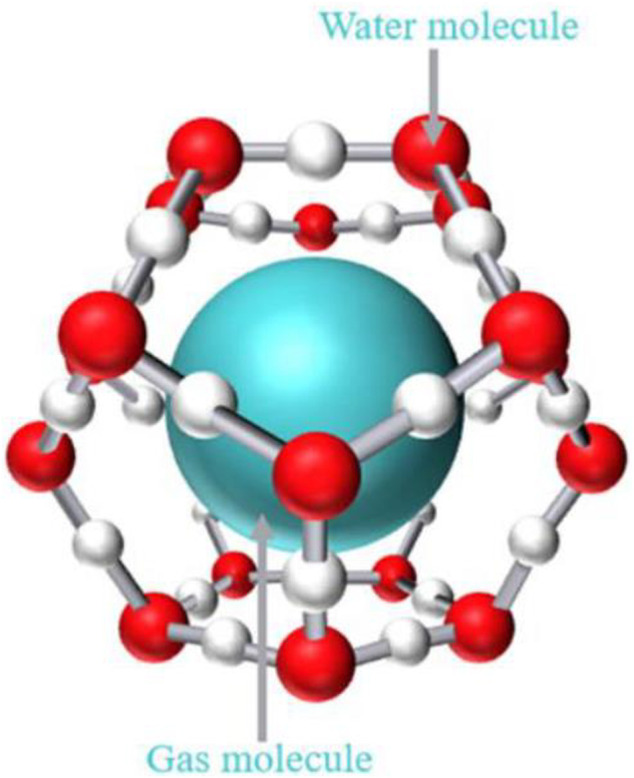
Schematic diagram of gas hydrate (Fu et al., 2022).

Gas hydrate is a clathrate compound, which is a cage-shaped crystal formed by small gas molecules and water at a certain temperature and pressure (Figure 6). The host water molecules form cage frame structures through hydrogen bonds, and gas molecules enter the cage structure to interact with the host molecules through van der Waals force to obtain a relatively stable non-stoichiometric crystal. There are three kinds of hydrate crystal structures discovered so far, which are type I, type II and type H (Warzinski and Holder, 1998). The separation of compound gas mixture is to use different gases to form hydrates with different degrees of difficulty, so as to achieve the separation effect.

The significant advantages of hydrate based gas separation (HBGS) technology are large gas storage capacity, reversibility of hydrate formation and decomposition processes. Therefore, the hydrate based gas separation technology has been considered as a promising CO_2_ capture technology (Li et al., 2024). During the capture process, the CO_2_ molecules are small and non-polar molecules, which results in the CO_2_ molecules being conducive to occupying the large cage. Compared with remaining gases in the mixture, the phase equilibrium pressure of CO_2_ hydrate at room temperature is lower than 10 MPa (Hassanpouryouzband et al., 2020), resulting in a significantly lower formation pressure of CO_2_ hydrate, which makes it have a higher affinity for the occupation of suitable cages in the hydrate crystal. The pure CO_2_ gas can be collected by dissociating the formed CO_2_ hydrate cage during the release process, while the water is returned to the liquid.

### 2.2 Phase equilibrium conditions of gas hydrate

The thermodynamic properties of hydrates have important research value in the field of oil and gas development and utilization, and the phase equilibrium conditions of hydrates are the key factors for the formation of hydrates. In the process of petroleum development and processing, mass transfer processes between phases are common, mainly including rectification, extraction, absorption, etc. When two phases are in contact, material exchange occurs between phases until the temperature, pressure, composition and other properties of each phase no longer change, and this state is the phase equilibrium state (Guo, 2002). When the separation of gas mixture based on hydrate method is conducted, due to the difference in the affinity between different guest molecules and water, one gas is enriched in the hydrate phase, and other gases enter the gas phase, and then the gas is recovered through hydrate decomposition (Sun et al., 2023). Common thermodynamic models used to predict hydrates include vdW-P model and Chen-Guo model. The vdW-P model derives the thermodynamic model of natural gas hydrate on the basis of the Langmuir gas isotherm adsorption theory (Naghibzade et al., 2015). The Chen-Guo model proposed a two-step mechanism for the formation of gas hydrate: 1) the quasi-chemical reaction process of forming basic hydrate; 2) the adsorption process of smaller gas molecules in the linked cavities of basic hydrate (Chen and Guo, 1998). This model establishes a simpler hydrate model, and in the temperature range from 273.4 K to 290.15 K, Chen-Guo model is more accurate than vdW-P model in predicting hydrate phase equilibrium (Zhang F Y et al., 2022).

In the CO_2_ capture process, CO_2_ is usually separated from the gas mixture. For multi-component mixed systems, grasping the phase equilibrium conditions is the key factor to achieve gas separation. Lee et al. (2016) used the state equation to simulate the phase equilibrium of carbon dioxide-rich mixed gas hydrates. This method is also applicable to the mixed gases including methane, ethane, propylene and nitrogen, and can be used for the calculation of two-phase and three-phase equilibrium systems. Sun et al. (2023) studied the mixing of three impurity gases (i.e., H_2_S, N_2_O, SO_2_) with CO_2_ gas at different concentrations. By comparing the hydrate phase abundance ratio and the mole fraction of CO_2_, it was found that impurity gases can be effectively eliminated at low concentrations. After separation, as the concentration of impurity gas increases, the abundance ratio and mole fraction of CO_2_ in the hydrate phase decrease significantly, which promotes the formation of CO_2_ hydrate to a certain extent. When the best concentration of impurity gas is 5%. One advantage of the hydrate method for separating CO_2_ gas is that the obtained CO_2_ hydrate can be used as a method for storing CO_2_. To ensure that CO_2_ could be stably stored in the hydrate state for a long time, it is vital to investigate the phase equilibrium conditions of CO_2_ hydrate. Kyung et al. (2014) studied the influence of marine environmental factors on the phase equilibrium of CO_2_ hydrate, obtained the three-phase equilibrium of CO_2_ hydrate in the presence of electrolytes, soil minerals and common organic matter in the seabed environment. The change of thermodynamic equilibrium conditions provides an important reference value for the seabed storage of CO_2_.

### 2.3 Enhanced methods for gas mixture separation based on hydrate technology

#### 2.3.1 Gas hydration separation in pure water system

**FIGURE 7:**
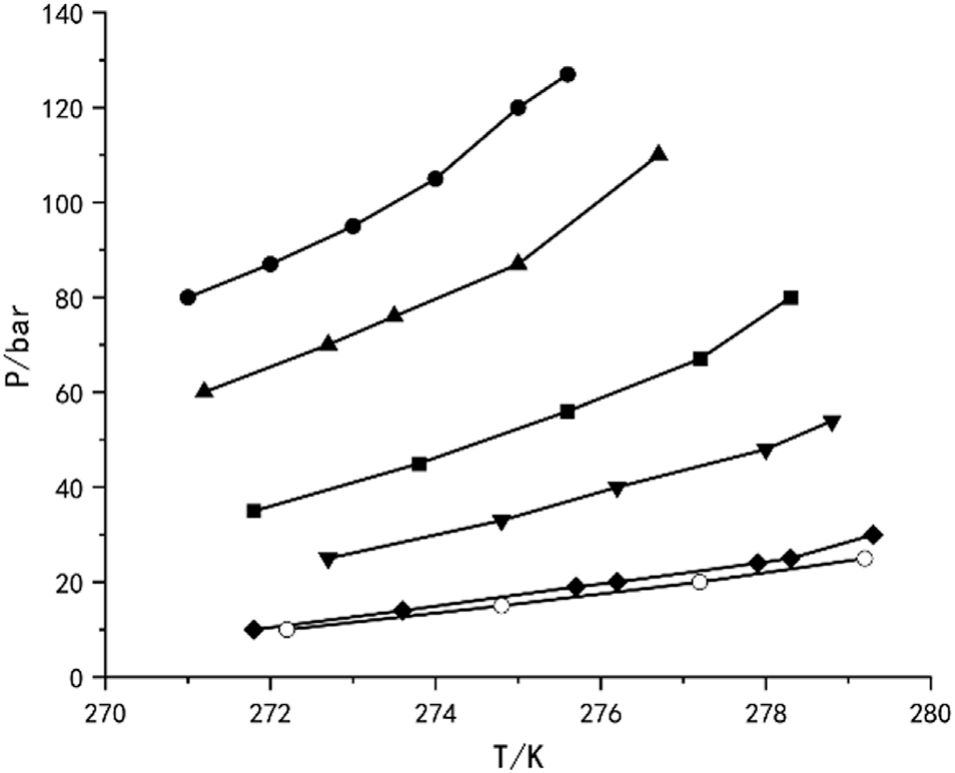
Hyrate phase equilibria for ternary N_2_+CO_2_+H_2_O mixturs of various CO_2_ compositions in 30.0 nm silica gel pores,and pure CO_2_+H_2_O mixture in bulk water:10 mol% CO_2_(●), 17 mol% CO_2_(▲), 35 mol% CO_2_(■), 50 mol% CO_2_(▼), 100 mol% CO_2_(◆), and 100 mol% CO_2_ in bulk water (○).

The research of separating various gas mixtures based on hydrate phase equilibrium principle has been relatively mature. As early as 2006, Park et al. (2006) measured the hydrate phase equilibrium of CO_2_-N_2_-H_2_O ternary system in silica gel pores. As shown in Figure 7 (Park et al., 2006), at a specific temperature, the H-Lw-V three-phase equilibrium curve moves to high pressure with the decrease of CO_2_ concentration in gas phase. Solid-state nuclear magnetic resonance spectrum shows that the mixed hydrate structure is SI type, and CO_2_ molecules mainly occupy 5^1,2^6^2^ cages. After three cycles of hydrate formation and dissociation, the CO_2_ concentration in the product can be as high as 96%. In 2016, Wang et al. (2016) studied the multistage separation of CH_4_/CO_2_ gas mixture based on hydrate method, which was carried out under different initial pressures. When the initial pressure is 4, 5 and 6 MPa, the proportion of CH_4_ increases from 72.24% to 97.22% and 97.14% after 4, 5 and 7 stage separation, respectively. The above research have confirmed the feasibility of gas mixture separation by hydrate method. However, the high operating pressure and low temperature seriously hindered the industrial development of this technology.

Different from pure component gases, the phase equilibrium of the mixed system is greatly affected by the gas composition, especially the mixed system containing CO_2_ (Nguyen et al., 2022). CO_2_ is easily soluble in water, and the composition in the equilibrium state is actually lower than the composition of the raw gas. The water-to-gas molar ratio affects the hydrate phase equilibrium by affecting the equilibrium gas composition, thereby affecting gas separation. Hydrate formation kinetics and separation efficiency are also one of the key factors in gas separation, which are directly related to the efficiency of HBGS process (Kim and Sa, 2023). In addition, the hydrate method also faces the problems of how to decrease energy consumption and improve separation selectivity, hydration rate, efficiency and hydrate stability. Recent years, researchers have studied the mechanical and chemical additives methods to enhance hydrate technology, and have achieved some success (Table 2).

**TABLE 2:** Enhanced methods for gas mixture separation based on hydrate technology.

Enhanced methods		Characteristics	References
Chemical additives	Kinetic promoter	Achieved by increasing the difference of hydration rate and induction time between guest molecules	Zhan G et al. (2022)
	Thermodynamic promoter	Achieved by temperature and pressure	Zheng et al. (2019)
	Nanoparticle additives	Promote mass transfer by enhancing gas-liquid disturbance and reduce the surface tension of gas-liquid interface, increase the dissolution rate of CO_2_	Cheng et al. (2021)
Mechanical reinforcement	Stirring	The gas-liquid contact area is greatly increased, which makes the hydrate formation rate improve rapidly	Xiao et al. (2018)
	Bubbling	Achieved by utilizing the countercurrent flow between gas and slurry in a bubble tower and the continuous updating of the gas-liquid contact interface	Lv et al. (2012)
	Spraying	Without stirring device, the tiny droplets move at a high speed under high pressure jet and react with gas molecules to form hydrate	Murakami et al. (2009)
Hydrate membrane technology		Achieved by the different permeability of different gas molecules through the membrane	Fan et al. (2020)

#### 2.3.2 Strengthening hydrate technology with chemical additives

##### 2.3.2.1 Kinetic promoter

There are two main ways to strengthen hydrate technology: kinetic strengthening and thermodynamic strengthening. Both of them strengthen hydration technology by increasing the dynamic and thermodynamic differences between different gas molecules. The greater the difference has, the better the separation effect. Kinetic enhancement is achieved by increasing the difference of hydration rate and induction time between guest molecules. In the current hydrate research experiments, kinetic enhancement is generally achieved by adding kinetic promoters. There are various chemical reagents that can be used as kinetic hydrate promoters, e.g., some amino acid and surfactants, which can form micelles with solubilization in aqueous solution and promote separation. Zhang G et al. (2022) studied the capture of CO_2_ by different kinds of amino acid aqueous solutions through microchannel mass transfer, and found that all kinds of amino acid salt solutions have good capture effect on CO_2_, with sodium glycine aqueous solution has the best capture effect. Deng et al. (2023) added fluorinated graphene with superhydrophobic nanostructure and surfactant sodium dodecyl sulfate (SDS) into the aqueous solution to promote the formation of CO_2_ hydrate. The results shows that graphene with superhydrophobic nanostructure can effectively promote the nucleation of CO_2_ hydrate and realize the continuous growth of hydrate. SDS can excellently improve the gas-liquid mass transfer efficiency and promote the dissolution of CO_2_. Liu et al. (2022) compared the promoting effect of SDS and L-methionine (L-Met) on CO_2_ hydrate formation and found that L-Met (0.1 wt%) promoted CO_2_ hydrates formation significantly with a gas uptake in CO_2_ hydrate five times more than SDS at the same concentration. The combination of fluorinated graphene and SDS greatly promotes the carbon storage performance of hydrate. In addition to the above two kinds of common kinetic promoters, graphene oxide (GO) has been studied in CO_2_ hydrate formation as the new kinetic promoter. Wang Y J et al. (2024) investigated the significant role of graphene oxide (GO) in enhancing the dynamic behaviors of post-combustion CO_2_ via the hydrate formation and found that the induction of GO increased the amount of gas transfer, and shortened the induction time for the hydrate nucleation at the gas-liquid interface. In the latest research, researchers tried to combine kinetic promoters with other additives, which can not only effectively promote hydrate formation and improve gas separation efficiency, but also make up for the shortcomings of traditional single kinetic promoters. Li et al. proposed an innovative idea of coupling Mg with amino acids to promote the formation of CO_2_ hydrate (Li et al., 2024). Experiments verified that the corrosion of Mg assisted hydrate nucleation, greatly shortened the time of CO_2_ hydrate nucleation, and proved that this method of promoting hydrate formation by coupling Mg with amino acids can also be applied to flue gas separation and hydrogen storage. Liu et al. tried to combine L-methionine, an environmentally friendly kinetic promoter, with low-dose tetrahydrofuran (THF), a thermodynamic promoter, and confirmed the synergistic mechanism of the coupling of kinetic and thermodynamic promoters in the process of CO_2_ hydrate formation, which provides guidance for the development of new environmentally friendly promoters (Liu et al., 2023).

##### 2.3.2.2 Thermodynamic promoter

**FIGURE 8:**
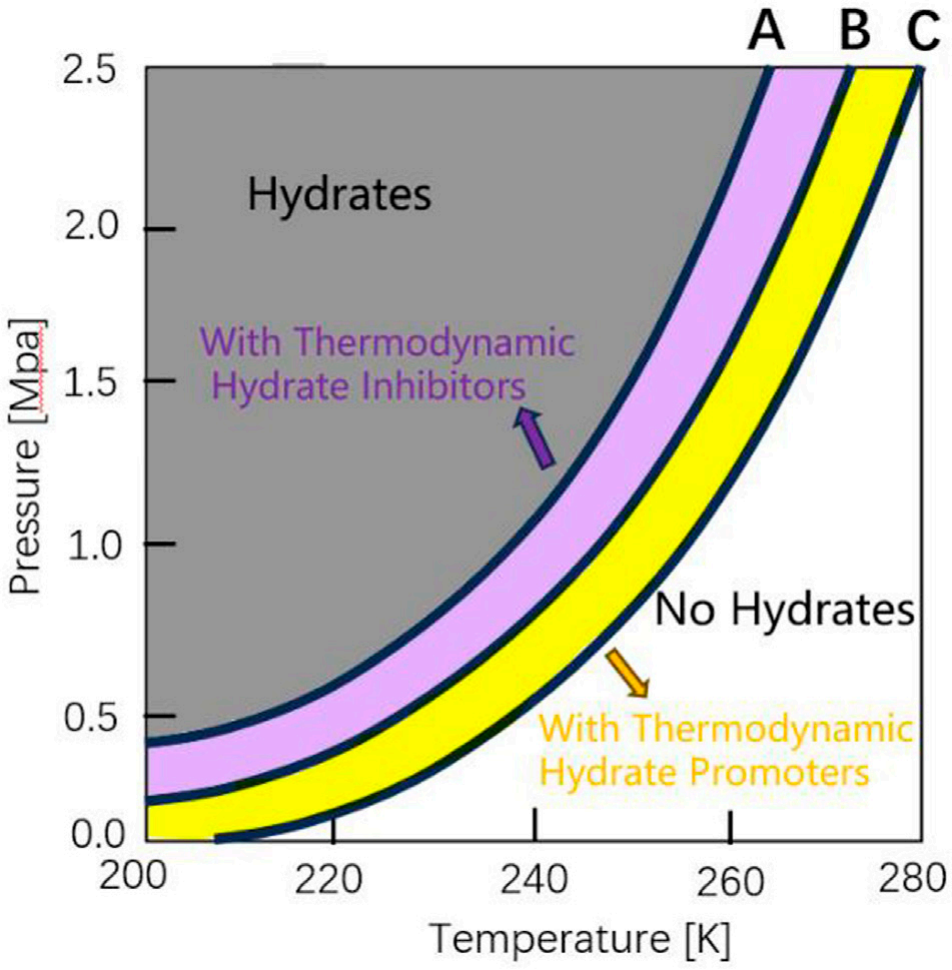
Example of hydrate equilibrium no THI/THP, curve **(B)** shift as a result of the addition of thermodynamic hydrate inhibitors (THIs, curve **(A)** or thermodynamic hydrate promoters (THPs, curve **(C)**.

Thermodynamic enhancement is achieved by temperature, pressure and other aspects. Thermodynamic promoters (e.g., THF (tetrahydrofuran), TBAB (tetra-n-butyl ammonium bromide), CP (cyclopentane), etc.) are usually added to enhance the separation effect by increasing the phase equilibrium difference between different gases (Zheng et al., 2019). Figure 8 (Majid et al., 2021) is a graph of hydrate equilibrium movement caused by adding thermodynamic hydrate inhibitor and promoter. The hydrate equilibrium curve depends on the gas composition and the promoter concentration. Promoter molecules participate in hydrate formation and are trapped by hydrate cages at low or high pressure, thus reducing the equilibrium conditions for hydrate formation. Yang et al. (2017) carried out the formation and dissociation characteristics of CO_2_/N_2_ hydrate under the condition of 19% THF, and found that 19% THF can promote hydrate formation by increasing hydrate formation rate and reducing equilibrium conditions. This discovery also confirmed that adding hydrate thermodynamic promoter makes hydrate formation conditions milder. Hydrate thermodynamic promoter can moderate the pressure condition as well as the temperature condition of hydrate formation. Zhou et al. (2018) measured the phase equilibrium conditions of CO_2_ hydrate formation with the TBAB concentration of 0.1–4.0 mol% at the pressure of 1.4–4.5 MPa. The results showed that when TBAB was added, the equilibrium temperature at constant pressure shifts 1–14 K to a milder condition. Since the thermodynamic promoter is directly involved in Yu et al. (2020) hydrate formation, many kinds of hydrates will be formed when separating the gas mixture. Yu et al. analyzed the separation effect of CP with different volume concentrations and gas-liquid ratios on CO_2_ in coal gasification combined cycle syngas, and the highest recovery ratio of CO_2_ was 98.8%. Meanwhile, the development of new separation technology based on the thermodynamic characteristics of hydrate has also been a hot research topic in recent years. Chen J L et al. (2020) proposed a new method of hydrate thermal-mass coupling, which uses the enthalpy of formation and dissociation of TBTA (tert-butyl trichloroacetylidene ester)/CO_2_ hydrate to reduce the energy consumption of CO_2_ separation, and explores the continuous separation of CO_2_, thus solving the problem of difficult heat transfer in gas-liquid mixed system in the traditional hydrate separation process.

##### 2.3.2.3 Nanoparticle additives

Besides the thermodynamic and kinetic additives mentioned above, nanoparticles can also promote the formation of hydrate. Nanoparticles have good thermal conductivity, and can promote to mass transfer by enhancing gas-liquid disturbance during hydrate formation (Cheng et al., 2021). In addition, the high specific surface area of nano-materials can reduce the surface tension of gas-liquid interface, increase the dissolution rate of CO_2_ and promote the formation of hydrate (Nashed et al., 2018). Khanmohammadian et al. (Khanmohammadian et al., 2023) used silica nanoparticles as promoter to separate CO_2_ from CO_2_/CH_4_ mixed gas in the presence of potassium hydroxide. The research shows that silica nanoparticles can promote the consumption, separation and recovery of mixed gas. Besides nonmetallic nanoparticles, metal nano-particles can also be used as hydrate promoters. Said et al. (2020) prepared an aluminum nano-fluid for CO_2_-CH_4_ hydrate formation process. The results showed that the added aluminum nano-particles can enhance gas dissolution and promote hydrate formation, and the best gas capture effect could be achieved at 0.3 wt%.

#### 2.3.3 Mechanical reinforcement

In addition to the chemical promotion methods mentioned above, there are also physical promotion methods during the hydrate formation process, namely, mechanical reinforcement, including stirring, bubbling and spraying.

##### 2.3.3.1 Stirring

**FIGURE 9:**
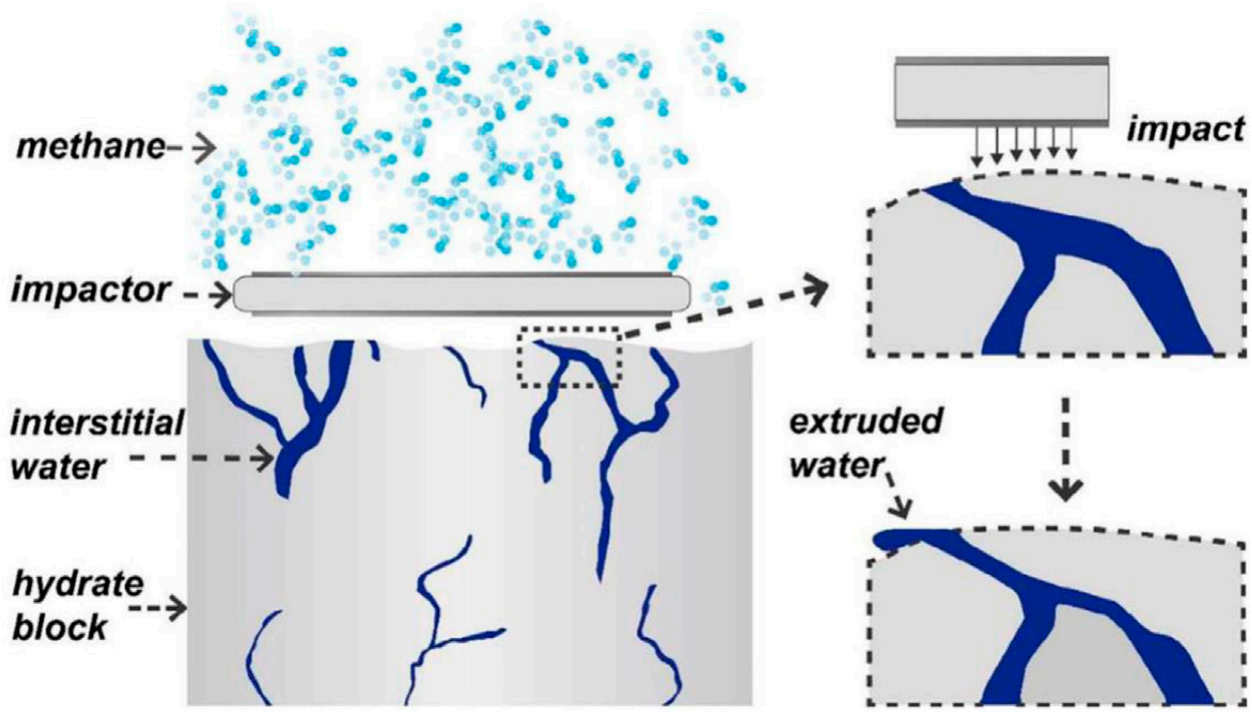
Sketch diagram of extruding interstitial water by reciprocating impact after bulk hydrate has formed.

Stirring is the most common mechanical promotion method. In the stirred system, the gas-liquid contact area is greatly increased, which makes the hydrate formation rate improve rapidly. As early as 2007, Hao et al. (2007) compared the temperature and reaction rate of methane hydrate synthesis with or without stirrer, and confirmed that intermittent stirring can enhance the heat and mass transfer performance of hydrate compound form process, effectively shorten the hydrate induction time. The suitable stirring speed at 5 MPa pressure is 320 rpm and the stirring time is 30 min, which provides a reference idea for further improving the storage capacity and formation rate of hydration process. However, with the formation of hydrate, the viscosity of slurry increases, which hinders the rotation of the stirring paddle (Fidel-Dufour et al., 2006). In the experiment of Linga et al. (2012) the hydrate in the stirring tank grew rapidly in the first 3 hours. And with the increase of hydrate amount, mixing becomes more and more difficult. The reaction stops after 60 h, and the final hydrate conversion ratio is only 74%. In order to solve this problem, in recent years, a new mechanical method, i.e., reciprocating impact method, has been developed. Reciprocating impact can make hydrate grow multiple times, promote the hydrate formation rate, and improve gas absorption amount. Xiao et al. (2018) used reciprocating impact instead of traditional stirring to promote hydrate formation by continuously impacting hydrate blocks with high pore moisture content, as shown in Figure 9. In the reciprocating impingement reactor, the water conversion ratio reaches more than 80% within 4 h, which is much higher than that of conventional stirring.

##### 2.3.3.2 Bubbling

**FIGURE 10:**
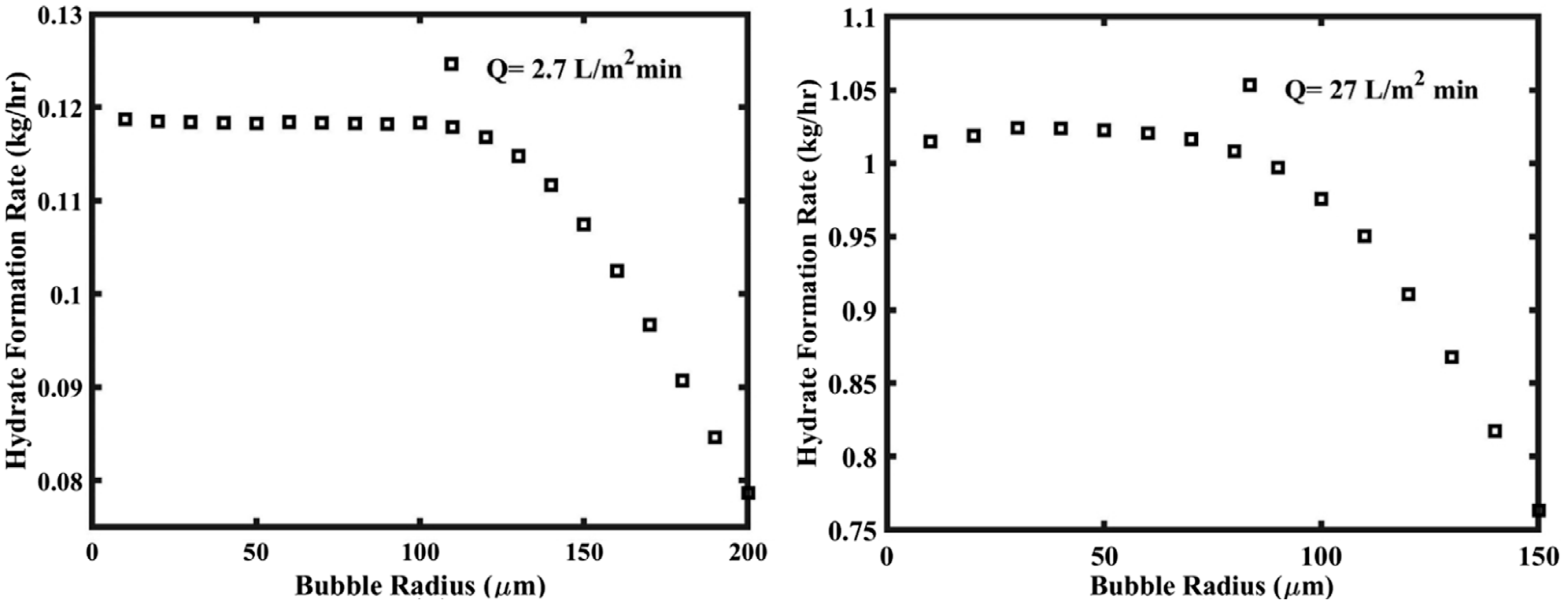
Influence of bubble size on hydrate formation rate for two gas flow rates.

Bubble column is a traditional industrial reactor used in gas-liquid reaction system. In industry, multi-stage separation of gas mixture is usually achieved by utilizing the countercurrent flow between gas and slurry in a bubble tower and the continuous updating of the gas-liquid contact interface. Lv et al. (2012) developed a large bubble column reactor in the industrial production of methane hydrate and the reactor has the following three characteristics. First, it does not need water circulation and saves the energy consumed by pumping water. Second, the gas is automatically introduced into water under the action of pressure difference. Last but not least, bubbles rise for a long time in the liquid phase, and hydrate is almost completely formed. The bubble column reactor effectively increases the gas-liquid contact area and significantly improves heat and mass transfer (Lang et al., 2010). Due to the movement of bubbles, the formation rate of hydrate on its surface is relatively high, the generated hydrate shell is not easy to break, and bubbles with hydrate shell cannot merge into large bubbles, which hinders the further formation of hydrate (Luo et al., 2007). According to this, Kar and Bahadur (2023) simulated the formation process of CO_2_ hydrate in bubble column, and the results are shown in Figure 10. Bubbles with diameter less than 100 μm were the key to improve the growth rate of hydrate, and increasing the reactor pressure could further improve the maximum theoretical separation efficiency of gas mixture.

##### 2.3.3.3 Spraying

**FIGURE 11:**
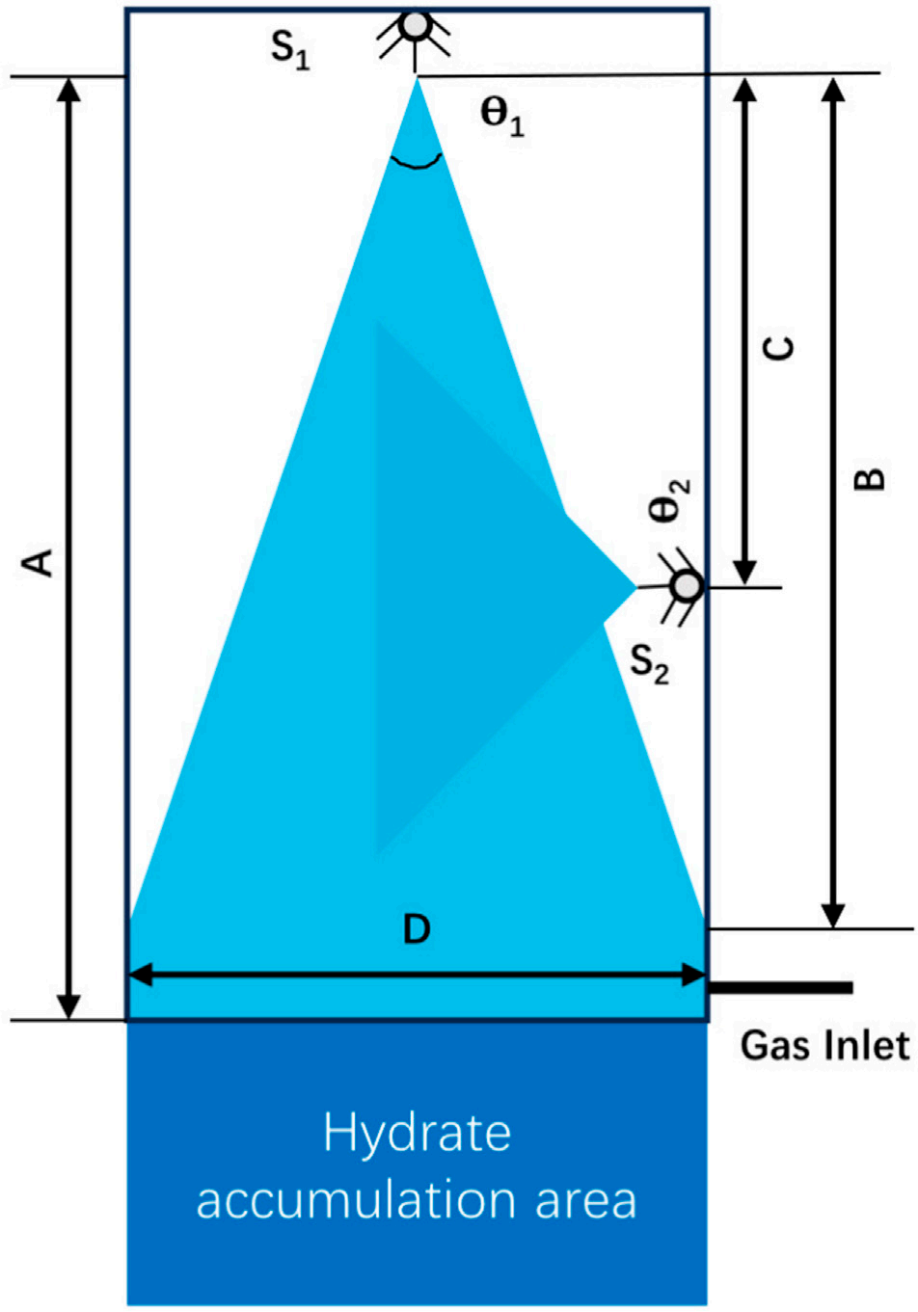
The geometry of main and quench spray nozzles. S_1_: Main spray nozzle with θ_1_ narrow spray angle, S_2_: Quench spray nozzle with θ_2_ wide spray angles, A: Gas dominate length, B: No wall contact length, C: Quench distance from main spray.

Spray reactor is another common reactor for gas-liquid reaction system, which increases the gas-liquid contact area by spraying water into the continuous gas phase region (Rehman and Lal, 2022). Without stirring device, the tiny droplets move at a high speed under high pressure jet and react with gas molecules to form hydrate. Murakami et al. (2009) carried out laboratory experiments on hydrate formation by using jet impingement device, which proved for the first time that jet device could be used in hydrate formation process. However, due to the existence of pressure difference, hydrate particles will grow in the gas flow loop, resulting in pipeline blockage. Li et al. (2010) explored the factors of rapid formation of CO_2_ hydrate in spray device, and found that higher initial pressure, i.e., driving force of nozzle, is favorable for promote the formation of hydrate, boost hydrate particles changing from spherical to branched shapes, and the circulating water pipeline will not be blocked by spraying small hydrate particles into gas phase. But with the increase of driving force, the total heat produced by hydrate formation will also increase. In order to overcome the problem of rapid increase in temperature in the spray reactor caused by heat release during hydrate formation, Partoon et al. (2018) were inspired by the direct contact heat exchanger, improved the design of nozzle and added quenching pipeline in the spray reactor to alleviate the unstable heat distribution in the reactor, and higher heat transfer rate can be achieved by the direct contact between heat flow and cold flow (Figure 11).

#### 2.3.4 Hydrate membrane technology

Hydrate membrane technology is a new type of gas separation technology, which uses the different permeability of different gas molecules through the membrane to separate gases. The driving force for gas passing through hydrate membrane mainly comes from the pressure difference between the two sides of the membrane. The newly developed hydrate membrane technology directly uses aqueous solution itself to form films. Substances, e.g., cyclopentane (CP) and tetrahydrofuran (THF), can form hydrate without the participation of gas molecules when the temperature of their aqueous solution is higher than zero degrees Celsius. Compared with the previous membrane technology, it greatly speeds up the formation rate of hydrates, increases the effective area of the membrane, and improves the separation efficiency. In addition, cyclopentane and other chemical substances can form membranes under normal pressure, which reduces the energy consumption in the experimental process. Fan et al. (2020) introduced membrane separation technology on the basis of hydrate separation of carbon dioxide, and used porous media on the membrane to adsorb carbon dioxide, which greatly improved the capture rate of carbon dioxide gas. Zhang Y Q et al. (2022) separated H_2_ by using different diffusion rates of mixed gas components through THF hydrate membrane. The experimental results show that only H_2_ can pass through the clathrate hydrate membrane with the thickness of 5 mm, while CH_4_ and CO_2_ in the gas mixture are prevented.

## 3 Application of hydrate method to capture carbon dioxide

Carbon dioxide is an easily hydrated gas. When mixed with other gases, it can be captured by hydrate. Separation of carbon dioxide by hydrate method is mainly divided into two steps: 1) first, carbon dioxide enters the cage structure of water molecules under high pressure and low temperature to form hydrate; 2) the formed cage hydrate structure is broken at low pressure and high temperature, and carbon dioxide is released, thus realizing separation. During the above operation, other gases which are not easy to be captured by the cage structure of water molecules are separated after the end of the first process. At present, several kinds of gas mixture involved in the field of CO_2_ separation and capture mainly include natural gas mainly composed of CO_2_/CH_4_, syngas tail gas mainly composed of CO_2_/H_2_ and flue gas mainly composed of CO_2_/N_2_. Due to the difference of composition and properties of the gas mixture, separation conditions of CO_2_ by hydrate method are also different. In natural gas CH_4_/CO_2_, the hydration pressure of the difficult-to-hydrate gas CH_4_ is twice as high as that of the easily-hydrated gas CO_2_ at 273.15 K. In the syngas tail gas (H_2_/CO_2_ gas mixture), the hydration pressure of the difficult-to-hydrate gas H_2_ is 200 times that of the easily-hydrated gas CO_2_ at 273.15 K. And in the flue gas mainly composed of N_2_/CO_2_, the hydration pressure of difficult-to-hydrate gas N_2_ is 12 times that of easily-hydrated gas CO_2_ at 273.15 K (Chen Z et al., 2020). Hydrate formation pressure of different gases at 273 K are shown in Table 3 (Wang et al., 2013).

**TABLE 3:** Hydrate formation pressure of different gases at 273K.

Gas	H_2_	N_2_	O_2_	CH_4_	CO_2_
*P*/MPa	213	16.3	11.1	2.65	1.22

### 3.1 Capture of carbon dioxide from natural gas

CH_4_ hydrate is usually synthesized at high pressure and low temperature, which the formation pressure is much higher than that of CO_2_ hydrate. According to this property, CO_2_ in natural gas can be separated. Gambelli et al. (2019) analyzed the hydrate method for separating natural gas and synthesized CH_4_ hydrate and CO_2_ hydrate at different pressures, and obtained hydrates with extremely high yields, which confirmed the feasibility of CO_2_/CH_4_ gas separation by using the hydrate method. As early as 2009, some scholars studied the subject of purifying natural gas by hydrate method. The results showed that the CO_2_ content was reduced from 25% to 16%, which confirmed that gas hydrate method can be used to purify natural gas (Van Denderen et al., 2009). However, this process of separating CO_2_/CH_4_ gas mixture by hydrate crystallization is very difficult. To solve this problem, Ricaurte et al. (2013) studied the influence of additives on hydrate formation, and found that when THF and SDS were mixed, the hydrate formation rate was the highest. In recent years, the research on CO_2_ storage mainly focuses on replacing the natural gas hydrate in the seabed with CO_2_. When carbon dioxide gas is injected into natural gas reservoir and reacts with methane, it can not only obtain a large amount of methane gas, but also store carbon dioxide gas stably for a long time. The disadvantage of this method is that usually takes a long time and consumes a lot of energy, and further improvement is needed in the follow-up development.

### 3.2 Capture of carbon dioxide from the tail gas of synthetic gas

Synthesis gas is a common intermediate raw gas in the processes of chemical industry, which is usually produced under high pressure. Its main components are H_2_, CO, CH_4_ and CO_2_. Under normal pressure, H_2_ hydrate require extremely low temperature to maintain a stable state (Mao et al., 2002). Compared with H_2_ hydrate, the formation conditions of CO_2_ hydrate are milder. The addition of hydrate promoters is usually employed to realize the high selectivity of H_2_O/CO_2_ gas mixture. Research has found that the clathrate hydrates constructed by tetra-n-butylammonium bromide (TBAB) and H_2_O molecules can capture gas molecules. The small hydrate cages constructed by the anions of TBAB solution capture small molecular gas, while the cations enters the large cages (Shimada et al., 2003). Cai et al. (2023) established a prediction model for the thermodynamic conditions of hydrate formation in the TBAB-containing aqueous solution system, and carried out experimental verification. The predicted value obtained were in good consistency with the experimental data, providing a theoretical basis for gas separation. Fukumoto et al. (2015) studied the dissociation conditions of H_2_ + CO_2_ smiclathrate hydrate formed with TBAB, TBAC, TBAF, TBPB, and TBNO_3_ salts, and found that CO_2_/H_2_ gas mixture could reach a high selectivity under the condition of moderate salt concentration.

### 3.3 Capture of carbon dioxide from flue gas

The flue gas generated by the combustion of fossil fuels contains a large amount of CO_2_ gas. It is reported that the CO_2_ emitted by power plants accounts for about 41% of the global CO_2_ emissions (Khatib, 2012). One of the important ways to achieve the carbon peaking is to capture CO_2_ from flue gas. The pressure difference between CO_2_ and N_2_ to form hydrate is much smaller than that between CO_2_ and H_2_. Theoretically, it is not difficult to capture CO_2_ from flue gas (Wang et al., 2013). The separation of CO_2_ gas from the flue gas based on the hydrate method has become a highly promising technology at present, and the capture ratio of this separation process has been verified to reach 99% (Mondal et al., 2012). However, to achieve industrialization, it is necessary to solve the problem of huge operating costs caused by compressing the gas to a pressure that forms hydrates. Hassanpouryouzband et al. (2019) proposed a new method for capturing CO_2_, injecting flue gas directly into water-bearing or ice-bearing sediments, integrating CO_2_ capture into a simple process, and significantly reducing the cost of carbon capture and storage. The CO_2_ efficiency of this method can reach 92% under certain conditions, which proves the feasibility of the hydrate method to capture CO_2_ gas from flue gas. Since the formation conditions of N_2_ hydrate are more severe than CO_2_ hydrate, a new technology of simulation has been developed, which is usually used to imitate the hydrate formation process to obtain the detailed requirements of hydrate formation (Lu et al., 2022). Kan et al. (2021) developed a new numerical simulation method, investigating the technology of injecting CO_2_/N_2_ into the actual permafrost hydrate reservoir to realize natural gas collection and CO_2_ sequestration by continuous injection-production model. The relationship between the storage rate and the CO_2_ content and pressure in the injected gas provides a basis for the practical application of CO_2_ storage and natural gas extraction. Zhang G et al. (2022) simulated the effects of different concentrations of dodecyl ammonium chloride (DTAC) solutions on the formation of CO_2_/N_2_ hydrate. Based on the simulation results, the higher the concentration of DTAC, the faster the hydrate formation rate, which lay a foundation for improving the carbon capture kinetics and separation performance based on hydrate.

### 3.4 Carbon capture and desalination

In recent years, with the continuous growth of the world population, the shortage of water resources and the high concentration of CO_2_ in the atmosphere have increasingly become a “double crisis” with great threat worldwide (Gautam et al., 2022). Carbon capture and seawater desalination based on hydrate have emerged as the times require. Industrially produced CO_2_ gas and seawater are injected into the hydrate reactor together. Under certain temperature and pressure conditions, water molecules form a cage structure around the CO_2_ molecules to generate hydrates. The hydrate dissociates under certain conditions to obtain CO_2_ gas and freshwater (Babu et al., 2020). However, factors such as the slow kinetics of hydrate formation have hindered the development of this technology. Gautam et al. found that salinity can inhibit the formation of hydrates, and adding thermodynamic promoters to the system can effectively increase the rate of hydrate formation (Gautam et al., 2022). This discovery provides a new insight for integrated carbon capture and seawater desalination of hydrates. Abulkhair et al. experimentally studied the effect of CO_2_-containing mixed gas hydrate formation on seawater desalination (Abulkhair et al., 2023). The results showed that the mixed gas hydrate formation can be used to treat produced water with low energy consumption according to the water recovery rate, removal efficiency, conductivity and other results. Montazeri et al. proposed the use of CO_2_ nanobubbles (NBs) as a sustainable kinetic promoter for the hydrate desalination process (Montazeri et al., 2024). The results confirmed that the memory effect of CO_2_ NBs played an important role in the desalination and effectively improved the desalination rate and ion removal rate, have provided ideas for further development of seawater desalination with natural salt solutions. These studies have shown that hydrate carbon capture and seawater desalination technology has broad application prospects. However, this technology still has problems such as high energy consumption and high cost. If it is to be applied to large-scale industrialization, it still needs to overcome some challenges.

## 4 Conclusion and prospect

In this paper, several common methods of CO_2_ capture are summarized, among which the capture of CO_2_ based on hydrate has become a promising technology because of its low cost and environmental friendliness. Moreover, we review the effect of various hydrate promoters on the hydrate formation process, e.g., kinetic promoters, thermodynamic promoters and nanoparticle additives. The reaction device is also crucial to the capture of CO_2_ based on hydrate method. Stirring, bubbling and spraying can promote hydrate formation. CO_2_ capture in industry is mainly carried out from three aspects: natural gas, syngas tail gas and flue gas. With the further implementation of the carbon emission policy, the study of capturing carbon dioxide will continue to intensify in the future. While reducing carbon dioxide emissions from the source, finding a simpler, more economical and environmentally friendly method of capturing CO_2_ requires researchers to invest more efforts. It is believed that with the improvement of science and technology, the problem of CO_2_ capture will eventually be properly solved.
